# Supplementary material for: Pyruvate Dehydrogenase Kinase 1 inhibition mediated oxidative phosphorylation enhancement in cartilage promotes osteoarthritis progression
Source: BMC Musculoskelet Disord. 2023 Jul 20;24:597. doi: 10.1186/s12891-023-06585-6 (PMC10357736; doi:10.1186/s12891-023-06585-6)
Supplement: Supplementary file 1 — Additional file 1. [file 12891_2023_6585_MOESM1_ESM.zip › KEGG.Gsea.1653622432464/KEGG_ARGININE_AND_PROLINE_METABOLISM.html]

Details for gene set KEGG\_ARGININE\_AND\_PROLINE\_METABOLISM[GSEA]

|  || Dataset | 55457\_collapsed\_to\_symbols.cls.cls #OA\_versus\_normal.cls.cls #OA\_versus\_normal\_repos |
| Phenotype | cls.cls#OA\_versus\_normal\_repos |
| Upregulated in class | OA |
| GeneSet | KEGG\_ARGININE\_AND\_PROLINE\_METABOLISM |
| Enrichment Score (ES) | 0.4269606 |
| Normalized Enrichment Score (NES) | 1.70587 |
| Nominal p-value | 0.005167959 |
| FDR q-value | 0.024720216 |
| FWER p-Value | 0.281 |
Table: GSEA Results Summary

  

Fig 1: Enrichment plot: KEGG\_ARGININE\_AND\_PROLINE\_METABOLISM      
 Profile of the Running ES Score & Positions of GeneSet Members on the Rank Ordered List

  

| SYMBOL | TITLE | RANK IN GENE LIST | RANK METRIC SCORE | RUNNING ES | CORE ENRICHMENT || 1 | GATM | glycine amidinotransferase [Source:HGNC Symbol;Acc:HGNC:4175] | 56 | 0.976 | 0.0452 | Yes |
| 2 | GOT1 | glutamic-oxaloacetic transaminase 1 [Source:HGNC Symbol;Acc:HGNC:4432] | 59 | 0.974 | 0.0946 | Yes |
| 3 | ACY1 | aminoacylase 1 [Source:HGNC Symbol;Acc:HGNC:177] | 86 | 0.937 | 0.1402 | Yes |
| 4 | AGMAT | agmatinase [Source:HGNC Symbol;Acc:HGNC:18407] | 147 | 0.866 | 0.1795 | Yes |
| 5 | P4HA2 | prolyl 4-hydroxylase subunit alpha 2 [Source:HGNC Symbol;Acc:HGNC:8547] | 258 | 0.779 | 0.2105 | Yes |
| 6 | P4HA1 | prolyl 4-hydroxylase subunit alpha 1 [Source:HGNC Symbol;Acc:HGNC:8546] | 833 | 0.560 | 0.1943 | Yes |
| 7 | ALDH2 | aldehyde dehydrogenase 2 family member [Source:HGNC Symbol;Acc:HGNC:404] | 1003 | 0.517 | 0.2074 | Yes |
| 8 | ALDH3A2 | aldehyde dehydrogenase 3 family member A2 [Source:HGNC Symbol;Acc:HGNC:403] | 1263 | 0.465 | 0.2109 | Yes |
| 9 | GLUD1 | glutamate dehydrogenase 1 [Source:HGNC Symbol;Acc:HGNC:4335] | 1279 | 0.462 | 0.2332 | Yes |
| 10 | GOT2 | glutamic-oxaloacetic transaminase 2 [Source:HGNC Symbol;Acc:HGNC:4433] | 1308 | 0.457 | 0.2542 | Yes |
| 11 | ASL | argininosuccinate lyase [Source:HGNC Symbol;Acc:HGNC:746] | 1318 | 0.455 | 0.2766 | Yes |
| 12 | ALDH18A1 | aldehyde dehydrogenase 18 family member A1 [Source:HGNC Symbol;Acc:HGNC:9722] | 1402 | 0.443 | 0.2926 | Yes |
| 13 | GAMT | guanidinoacetate N-methyltransferase [Source:HGNC Symbol;Acc:HGNC:4136] | 1418 | 0.441 | 0.3139 | Yes |
| 14 | GLUD2 | glutamate dehydrogenase 2 [Source:HGNC Symbol;Acc:HGNC:4336] | 1554 | 0.420 | 0.3247 | Yes |
| 15 | AOC1 | amine oxidase copper containing 1 [Source:HGNC Symbol;Acc:HGNC:80] | 1992 | 0.362 | 0.3091 | Yes |
| 16 | OAT | ornithine aminotransferase [Source:HGNC Symbol;Acc:HGNC:8091] | 2074 | 0.353 | 0.3207 | Yes |
| 17 | PYCR3 | pyrroline-5-carboxylate reductase 3 [Source:HGNC Symbol;Acc:HGNC:25846] | 2136 | 0.346 | 0.3335 | Yes |
| 18 | PYCR1 | pyrroline-5-carboxylate reductase 1 [Source:HGNC Symbol;Acc:HGNC:9721] | 2145 | 0.345 | 0.3504 | Yes |
| 19 | ALDH7A1 | aldehyde dehydrogenase 7 family member A1 [Source:HGNC Symbol;Acc:HGNC:877] | 2158 | 0.343 | 0.3669 | Yes |
| 20 | CPS1 | carbamoyl-phosphate synthase 1 [Source:HGNC Symbol;Acc:HGNC:2323] | 2178 | 0.341 | 0.3828 | Yes |
| 21 | MAOB | monoamine oxidase B [Source:HGNC Symbol;Acc:HGNC:6834] | 2192 | 0.339 | 0.3990 | Yes |
| 22 | CKM | "creatine kinase, M-type [Source:HGNC Symbol;Acc:HGNC:1994]" | 2403 | 0.314 | 0.3986 | Yes |
| 23 | ASS1 | argininosuccinate synthase 1 [Source:HGNC Symbol;Acc:HGNC:758] | 2466 | 0.307 | 0.4094 | Yes |
| 24 | CKMT2 | "creatine kinase, mitochondrial 2 [Source:HGNC Symbol;Acc:HGNC:1996]" | 2621 | 0.288 | 0.4120 | Yes |
| 25 | OTC | ornithine transcarbamylase [Source:HGNC Symbol;Acc:HGNC:8512] | 2628 | 0.287 | 0.4261 | Yes |
| 26 | ALDH9A1 | aldehyde dehydrogenase 9 family member A1 [Source:HGNC Symbol;Acc:HGNC:412] | 2795 | 0.270 | 0.4270 | Yes |
| 27 | LAP3 | leucine aminopeptidase 3 [Source:HGNC Symbol;Acc:HGNC:18449] | 3044 | 0.245 | 0.4201 | No |
| 28 | NOS1 | nitric oxide synthase 1 [Source:HGNC Symbol;Acc:HGNC:7872] | 4228 | 0.134 | 0.3349 | No |
| 29 | ARG2 | arginase 2 [Source:HGNC Symbol;Acc:HGNC:664] | 4476 | 0.115 | 0.3215 | No |
| 30 | CKMT1B | "creatine kinase, mitochondrial 1B [Source:HGNC Symbol;Acc:HGNC:1995]" | 5810 | 0.008 | 0.2182 | No |
| 31 | ODC1 | ornithine decarboxylase 1 [Source:HGNC Symbol;Acc:HGNC:8109] | 6153 | -0.016 | 0.1925 | No |
| 32 | DAO | D-amino acid oxidase [Source:HGNC Symbol;Acc:HGNC:2671] | 6252 | -0.024 | 0.1861 | No |
| 33 | ALDH1B1 | aldehyde dehydrogenase 1 family member B1 [Source:HGNC Symbol;Acc:HGNC:407] | 6551 | -0.046 | 0.1652 | No |
| 34 | PRODH | proline dehydrogenase 1 [Source:HGNC Symbol;Acc:HGNC:9453] | 6927 | -0.074 | 0.1398 | No |
| 35 | CKB | creatine kinase B [Source:HGNC Symbol;Acc:HGNC:1991] | 7358 | -0.106 | 0.1117 | No |
| 36 | SRM | spermidine synthase [Source:HGNC Symbol;Acc:HGNC:11296] | 7735 | -0.136 | 0.0894 | No |
| 37 | MAOA | monoamine oxidase A [Source:HGNC Symbol;Acc:HGNC:6833] | 8271 | -0.177 | 0.0568 | No |
| 38 | GLS | glutaminase [Source:HGNC Symbol;Acc:HGNC:4331] | 8423 | -0.187 | 0.0545 | No |
| 39 | SMS | spermine synthase [Source:HGNC Symbol;Acc:HGNC:11123] | 8884 | -0.223 | 0.0301 | No |
| 40 | ARG1 | arginase 1 [Source:HGNC Symbol;Acc:HGNC:663] | 9556 | -0.279 | -0.0080 | No |
| 41 | NOS3 | nitric oxide synthase 3 [Source:HGNC Symbol;Acc:HGNC:7876] | 9980 | -0.319 | -0.0247 | No |
| 42 | AMD1 | adenosylmethionine decarboxylase 1 [Source:HGNC Symbol;Acc:HGNC:457] | 10415 | -0.363 | -0.0400 | No |
| 43 | GLS2 | glutaminase 2 [Source:HGNC Symbol;Acc:HGNC:29570] | 11547 | -0.507 | -0.1023 | No |
| 44 | ALDH4A1 | aldehyde dehydrogenase 4 family member A1 [Source:HGNC Symbol;Acc:HGNC:406] | 12080 | -0.614 | -0.1125 | No |
| 45 | NOS2 | nitric oxide synthase 2 [Source:HGNC Symbol;Acc:HGNC:7873] | 12262 | -0.665 | -0.0928 | No |
| 46 | GLUL | glutamate-ammonia ligase [Source:HGNC Symbol;Acc:HGNC:4341] | 12527 | -0.762 | -0.0746 | No |
| 47 | SAT1 | spermidine/spermine N1-acetyltransferase 1 [Source:HGNC Symbol;Acc:HGNC:10540] | 12807 | -0.982 | -0.0465 | No |
| 48 | PRODH2 | proline dehydrogenase 2 [Source:HGNC Symbol;Acc:HGNC:17325] | 12847 | -1.055 | 0.0041 | No |
Table: GSEA details [plain text format]

  

Fig 2: KEGG\_ARGININE\_AND\_PROLINE\_METABOLISM      
 Blue-Pink O' Gram in the Space of the Analyzed GeneSet

  

Fig 3: KEGG\_ARGININE\_AND\_PROLINE\_METABOLISM: Random ES distribution      
 Gene set null distribution of ES for **KEGG\_ARGININE\_AND\_PROLINE\_METABOLISM**
